# Non-linear dose-response relationship between uterine artery pulsatility index and risk of preeclampsia in early pregnancy: A secondary analysis based on a nested cohort study

**DOI:** 10.1371/journal.pone.0317625

**Published:** 2025-01-16

**Authors:** Shaou Wang, Hao Dong

**Affiliations:** 1 Department of Ultrasound, The First People’s Hospital of Xiaoshan District, Xiaoshan Affiliated Hospital of Wenzhou Medical University, Hangzhou, Zhejiang, China; 2 Department of Radiology, The First People’s Hospital of Xiaoshan District, Xiaoshan Affiliated Hospital of Wenzhou Medical University, Hangzhou, Zhejiang, China; University of Calabria, ITALY

## Abstract

**Background:**

Previous studies have shown that higher uterine artery pulsatility index (UtA-PI) values in early pregnancy have predictive value for the risk of preeclampsia (PE). However, the sensitivity and specificity of this marker remain controversial. This study aims to explore further the association between UtA-PI in early pregnancy and the incidence of preeclampsia.

**Methods:**

A total of 5000 pregnant women who underwent prenatal examination and delivery at the same hospital were included in this nested cohort study. And the PI values of left and right uterine arteries of the subjects were obtained by transabdominal ultrasound using GE color doppler diagnostic device in early pregnancy (11–13 ^+ 6^ weeks), and finally the mean value of both was calculated and recorded as UtA-PI. Among them, 60 pregnant women developed preeclampsia and were randomly divided into a screening group (n = 12) and control group (n = 48) and matched with pregnant women who did not develop preeclampsia during the same period to form a subset for subsequent statistical analysis. A weighted multivariate logistic regression model was used to analyze the association between UtA-PI and PE. Additionally, the non-linear relationship between UtA-PI and the incidence of PE was examined using smooth curve fitting and a generalized additive model.

**Results:**

After adjusting for other variables, UtA-PI values were positively correlated with the incidence of PE, and the relationship showed a non-linear U-shaped relationship (inflection point 1.83).

**Conclusion:**

Our study showed a significantly increased risk of PE when UtA-PI exceeded 1.83. This provides a basis for clinicians to identify high-risk pregnant women early and implement timely intervention, which helps to reduce maternal and fetal complications and improve health outcomes.

## Introduction

Preeclampsia (PE) is a hypertensive disorder associated with pregnancy that usually presents with hypertension and proteinuria after 20 weeks of gestation. Globally, the incidence of PE ranges between 2% and 8% [1, 2], and the specific incidence varies by region from 0.2% to 9.2% [3]. PE can be divided into early onset (occurring before 34 weeks of gestation) and late onset (occurring after 34 weeks of gestation and close to before delivery) [4]. Without timely intervention, PE may progress to eclampsia, posing a threat to maternal and fetal health [5–7]. Therefore, early identification and management of patients with preeclampsia is essential. The uterine artery pulsatility index (UtA-PI) is an index of uterine artery hemodynamics assessed by ultrasound, which reflects the blood flow resistance of the uterine artery [8, 9]. During normal pregnancy, the uteroplacental circulation should present a "low-resistance vascular bed", which contributes to the well-developed placenta and the maintenance of pregnancy [10–12]. However, elevated resistance to uterine artery blood flow is typically indicated by high UtA-PI readings, and this is typically linked to aberrant physiological changes in the vasculature.

Previous studies have shown that increased resistance to uterine artery blood flow increases the risk of adverse pregnancy outcomes such as PE and fetal growth restriction(FGR) [13]. Some scholars have also pointed out that the UtA-PI value can be an important indicator for predicting PE. Mechanisms between abnormalities in uterine artery blood flow and the development of PE mainly involve impaired trophoblast invasion and subsequent placental dysfunction [14, 15]. High UtA-PI values indicate increased uterine artery resistance, suggesting insufficient remodeling of the maternal spiral arteries, which is essential for maintaining normal placental blood flow. This remodeling defect may lead to uteroplacental ischemia, thereby contributing to the development of PE disease [16–18]. In addition, the timing of Doppler examination plays a key role in predicting patients with PE, and it has been pointed out that measurement of UtA-PI in early pregnancy can be used as an independent predictor of early-onset PE [19, 20], but this view is more controversial, and there is no uniform consensus on its predictive efficacy.

The results of this study will aid in the early identification of high-risk pregnant women. As a result, we carried out a secondary analysis of data from a nested cohort to thoroughly examine the relationship between UtA-PI in early pregnancy and the incidence of PE. Based on the UtA-PI values, risk stratification of pregnant women can be achieved, enabling personalized management, early intervention, and improved maternal and neonatal outcomes. This approach also allows for more efficient allocation of medical resources, particularly in resource-limited settings [1, 21].

## Materials and methods

### Ethical approval

This study is based on a secondary analysis of a nested cohort study derived from *"Plasma SerpinA5 in conjunction with uterine artery pulsatility index and a clinical risk factor for the early prediction of preeclampsia"* by Yonggang Zhang et al., published in *PLOS ONE* on October 14, 2021. The study has been approved by the Ethics Committee of Shenzhen Longhua District Central Hospital, and all participants obtained written informed consent and were anonymized and numbered.

### Study population

**Fig 1:**
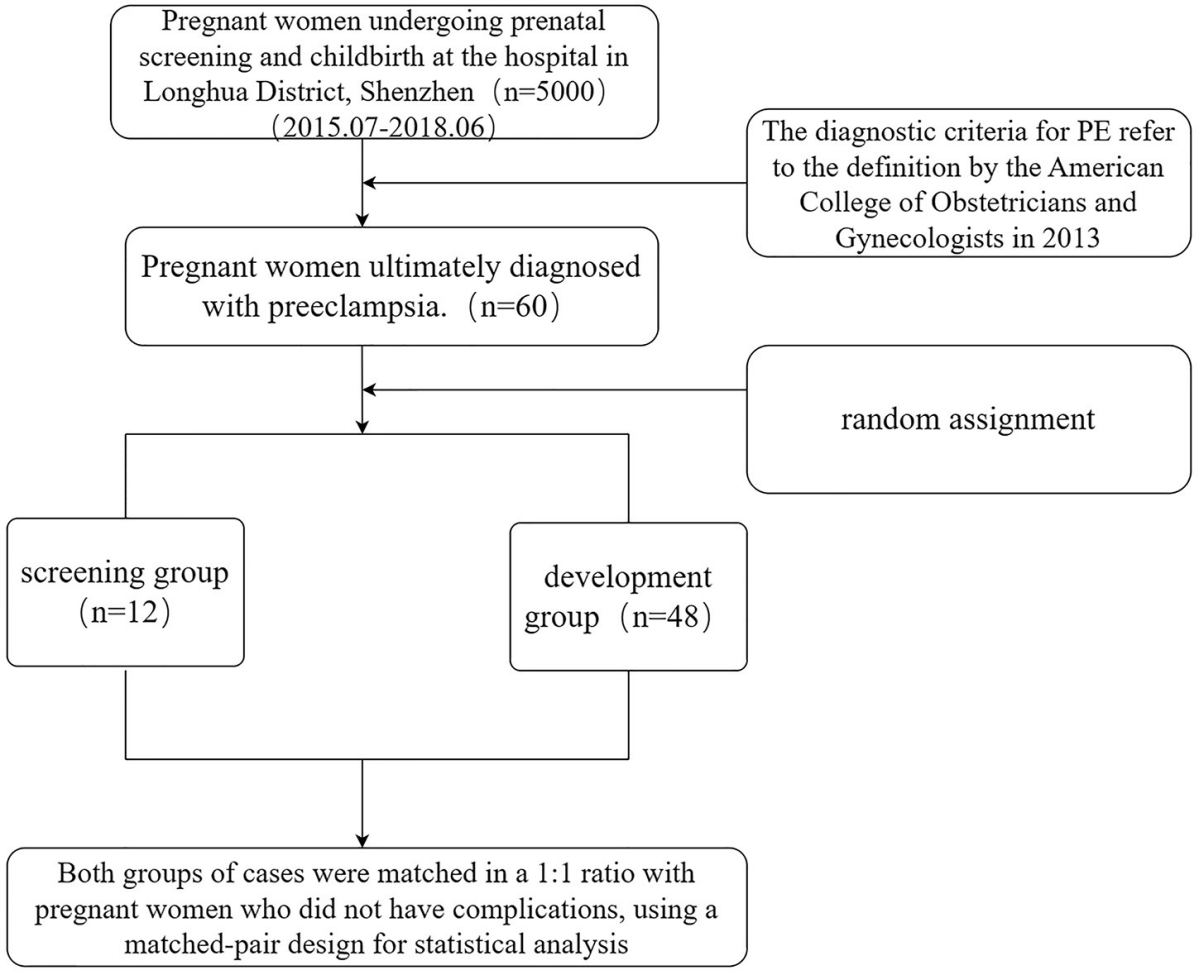
Flowchart of participant selection.

The study used a nested cohort design and was conducted in the obstetrics department of Longhua District Hospital, Shenzhen, China. The study cohort consisted of 5000 pregnant women who underwent prenatal screening and delivery at this hospital from July 2015 to June 2018. Participants who met the inclusion criteria were pregnant women who developed PE after a gestational age of more than 20 weeks. The diagnostic criteria for PE refer to the 2018 American College of Obstetricians and Gynecologists definition [22]: refers to the occurrence of hypertension and proteinuria after 20 weeks of gestation, or hypertension accompanied by other serious features, and the conditions for confirming hypertension are that blood pressure is measured twice at least 4 hours apart. Exclusion criteria included pregnant women with multiple pregnancies, stillbirths, gestational diabetes, uterine fibroids, or hyperthyroidism. In the initial cohort of 5000, 60 pregnant women were diagnosed with PE, constituting a subset sample. The 60 pregnant women with PE were then randomly divided into a screening group (12 pregnant women with PE), and a development group (48 pregnant women with PE), and both groups were matched 1:1 with pregnant women without complications for age, gestational age, and sampling date. This subset sample will be used to analyze in detail the clinical findings and factors associated with preeclampsia. Fig 1 shows the sample selection flowchart.

### Variables

#### Measurement of UtA-PI

Obtained by transabdominal ultrasonography using a GE color Doppler ultrasound device at 11–13 ^+ 6^ weeks of gestation. Participants lay in the supine position on the examination bed with a moderately filled bladder. The cervix was located, and the image was locally magnified. The probe was moved to one side of the cervix at the horizontal level, and color Doppler was used to display the ascending branch of the uterine artery. The pulsatility index (PI) value was obtained after acquiring typical uterine artery blood flow waveforms across three consecutive cardiac cycles. After locating the ascending branch of the contralateral uterine artery, the probe was transferred back to the cervix, where PI was measured. A 2 mm sample gate width and a <30° angle between the ultrasound beam and the blood flow were maintained. The mean PI value of both sides was calculated and recorded.

#### Measurement of mean arterial pressure (MAP)

Systolic blood pressure (SBP) and diastolic blood pressure (DBP) were obtained from the medical record system during the prenatal examination of pregnant women in early pregnancy (11 ~ 13 ^+ 6^ weeks) and calculated and recorded according to the formula MAP = DBP + (SBP-DBP)/3.

### Collection of clinical epidemiology data

Clinical risk factors were collected from the case system and prenatal and postnatal questionnaires, respectively. These included smoking history, pre-pregnancy body mass index (BMI), gestational age at delivery, presence of a family history of preeclampsia, 24-hour urine protein, gestational age at delivery, and neonatal birth weight.

### Statistical analysis

We utilized a variance estimation approach along with weighting to accommodate significant variation in the dataset. Weighted multivariate logistic regression models were used to analyze the association between UtAPI and PE. We employed weighted chi-square tests for categorical data and weighted linear regression models for continuous variables in order to find group differences. The non-linear association between UtAPI and PE was further studied using a generalized additive model and smooth curve fitting. After the nonlinearity was found, the inflection point of the relationship between UtAPI and PE was calculated using recursive techniques. On both sides of the inflection point, a two-segment linear regression model was used. A P value of less than 0.05 was deemed statistically significant for all analyses, which were carried out using EmpowerStats (http://www.empowerstats.com) and the R program (http://www.Rproject.org).

## Results

Table 1 presents the clinical features of each subject, categorized into low, middle, and high groups based on UtA-PI tertiles. There were 32 people in each group, for a total of 96 participants. Between the three groups, there was no statistically significant difference in delivery times (p = 0.867). Significant variations were noted in the clinical and cardiovascular markers, nevertheless. The results indicated that higher UtA-PI values were associated with more severe cardiovascular and clinical characteristics, including elevated blood pressure, increased proteinuria, lower newborn birth weight, and a higher proportion of PE cases.

**Table 1:** Baseline characteristics of participants (n = 96).

UtA-PI tertile	T1 (0.54~1.69)	T2 (1.71~2.09)	T3 (2.12~3.64)	P-value
N	32	32	32	
delivery (w)	36.19 ± 1.30	36.02 ± 1.59	36.00 ± 1.73	0.867
Systolic (mmHg)	132.78 ± 22.32	129.53 ± 23.09	149.16 ± 17.67	<0.001
Diastolic (mmHg)	86.50 ± 13.91	86.41 ± 15.57	98.16 ± 13.96	0.002
Proteinuria (g/24 h)	0.94 ± 1.98	0.47 ± 0.76	1.85 ± 2.20	0.009
Smoking(n)	0.00 ± 0.00	0.00 ± 0.00	0.00 ± 0.00	
Pre-pregnancy BMI (kg/m2)	25.05 ± 2.55	24.98 ± 2.53	25.89 ± 2.76	0.310
SerpinA5 (ng/ml)	0.13 ± 0.17	0.21 ± 0.25	0.22 ± 0.22	0.182
New-born weight (g)	3156.47±620.55	3130.66±674.56	2765.66±690.56	0.035
MAP(mmHg)	101.93 ± 15.82	100.78 ± 17.35	115.16 ± 14.17	<0.001
groups				<0.001
control groups(n, %)	21 (65.62%)	21 (65.62%)	6 (18.75%)	
preeclampsia (n, %)	11 (34.38%)	11 (34.38%)	26 (81.25%)	
family history				0.614*
No family history of preeclampsia(n, %)	31 (96.88%)	29 (90.62%)	31 (96.88%)	
Family history of preeclampsia(n, %)	1 (3.12%)	3 (9.38%)	1 (3.12%)	

Mean ± SD for continuous variables: the p-value was calculated by the weighted linear regression model. (%) for categorical variables: the p-value was calculated by the weighted chi-square test.

*: Fisher’s exact probability test.

The results of the multivariate regression analysis are shown in Table 2. UtA-PI was related to PE in a significant way in the unadjusted model, with an odds ratio (OR) of 4.34 (95% confidence interval [CI], 1.92 to 9.82; p = 0.0004). This suggests that for each 1-unit increase in UtA-PI, the likelihood of developing PE increased by 4.34 times. In the adjusted model, key factors such as SerpinA5 levels (ng/ml), pre-pregnancy BMI (kg/m^2^), and family history were adjusted. After adjustment, the association between UtA-PI and PE remained statistically significant. The adjusted OR was 3.42 (95% CI, 1.10 to 10.59; p = 0.0331), suggesting that even after accounting for potential confounders, each 1-unit increase in UtA-PI was related with a 3.42-fold increase in the likelihood of PE.

**Table 2:** The association between UtA-PI and preeclampsia.

Exposure	Non-adjusted	Adjust
UtA-PI	4.34 (1.92, 9.82) 0.0004	3.39 (1.04, 11.02) 0.0422

Non-adjusted model adjusts for: None.

Adjust model adjust for: SerpinA5 (ng/ml); Pre-pregnancy BMI (kg/m2); family history.

**Fig 2:**
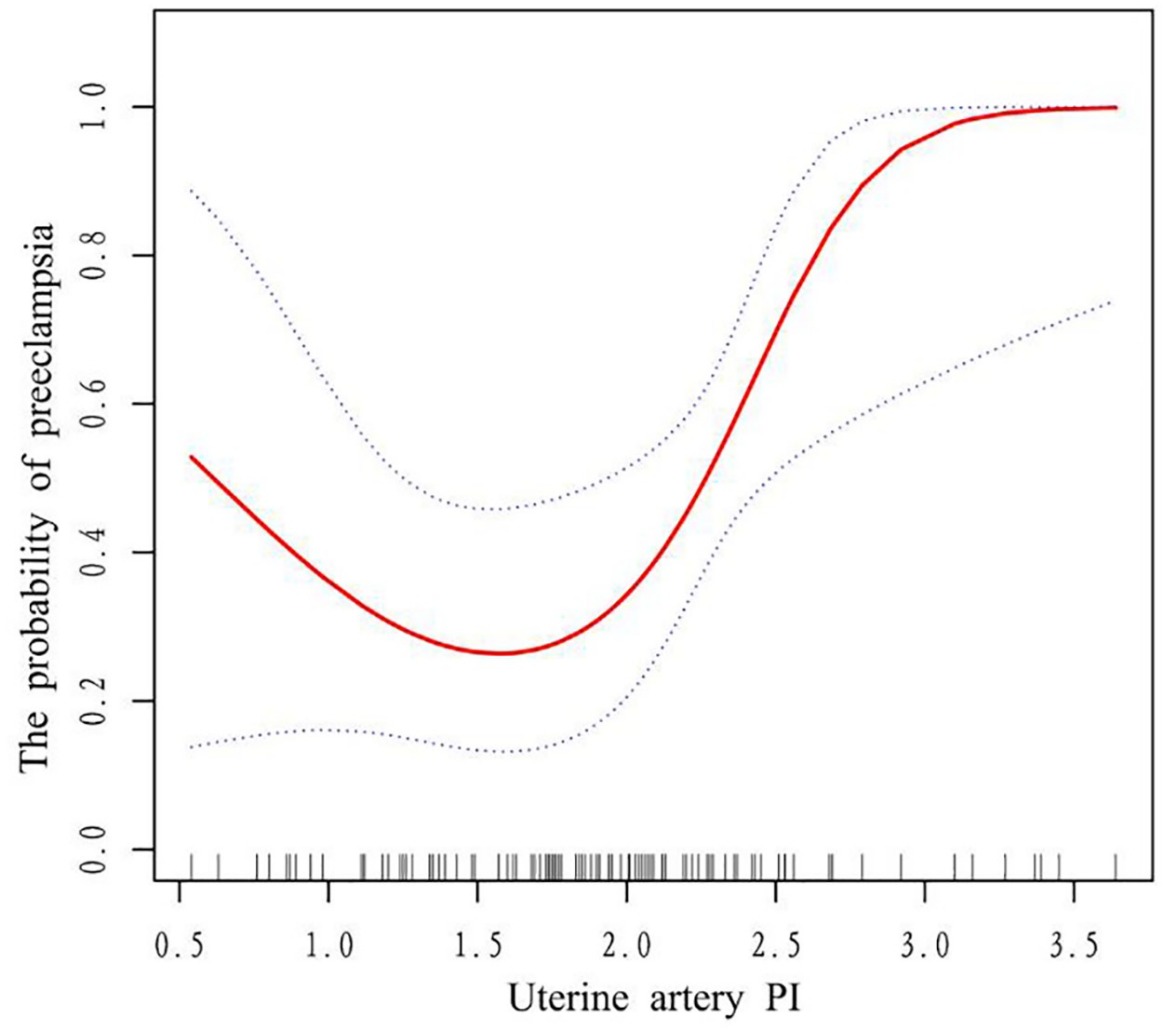
Association between UtA-PI and preeclampsia. Solid rad line represents the smooth curve fit between variables. Blue bands represent the 95% of confidence interval from the fit. Pre-pregnancy BMI (kg/m2); family history; SerpinA5 (ng/ml); MAP dichotomous were adjusted.

The non-linear association between UtA-PI and the incidence of PE was explained by the generalized additive model and smooth curve fitting, as illustrated in Fig 2. After performing additional threshold effect analysis, we discovered a non-linear association between UtA-PI and PE incidence (Table 3). We identified 1.83 as the inflection point of the U-shaped connection between UtA-PI and PE incidence using a two-segment linear model. For UtA-PI values less than 1.83, the OR was 0.27 (95% CI, 0.03 to 2.25; p = 0.2242), indicating no significant association between UtA-PI and PE. In contrast, for UtA-PI values greater than 1.83, the OR was 48.49 (95% CI, 2.55 to 923.82; p = 0.0235), suggesting that higher UtA-PI levels were significantly related with an increased risk of PE. The likelihood ratio test result yielded a p-value of 0.011, indicating that when compared to a normal linear model, the two-segment linear model fit the data better.

**Table 3:** Threshold effect analysis of UtA-PI and preeclampsia using piece-wise linear regression.

preeclampsia	OR (95% CI), p-value
Fitting model using standard linear mode	3.42 (1.10, 10.59) 0.0331
Fitting by the two-piecewise linear model	1.83
Inflection of UtPI	
UtPI < 1.83	0.27 (0.03, 2.25) 0.2242
UtPI >1.83	48.49 (2.55, 923.82) 0.0235
Log likelihood ratio	0.011

Pre-pregnancy BMI (kg/m2); family history; SerpinA5 (ng/ml); were adjusted.

## Discussion

So far, the pathogenesis of PE remains unclear, and there is no effective treatment. The condition can only be alleviated after the delivery of the placenta and fetus [23]. Therefore, improving the predictive ability of PE and finding effective predictors have particularly important realistic clinical significance. It was observed that a greater percentage of PE prevalence was substantially correlated with higher UtA-PI readings. Multiple regression analysis further confirmed that the odds of PE increased 3.42-fold with each unit increase in UtA-PI, even after adjusting for potential confounders such as SerpinA5 level, pre-pregnancy BMI, and family history, with an OR of 3.42 and a 95% CI of 1.10 to 10.59 (p = 0.0331). In addition, our study also found a non-linear U-shaped relationship between UtA-PI and the incidence of PE, and this critical inflection point was identified by threshold effect analysis, that is, when UtA-PI exceeded 1.83, it was significantly associated with the risk of PE, with an OR of 48.49 (95% CI: 2.55, 923.82, p = 0.0235), but below 1.83, with an OR of 0.27 (95% CI: 0.03, 2.25, p = 0.2242). This finding reveals a complex interaction between uterine artery blood flow and the onset of PE.

Normal blood circulation in the uterine arteries of the mother’s body facilitates the maintenance of the function of the placenta, thus ensuring the growth of the fetus. Several maternal factors such as higher body mass index, hypertriglyceridemia, heart rate, race, and use of antihypertensive medications can affect the impedance of the uterine arteries thereby altering the hemodynamics of the uterine arteries [24, 25]. Trophoblast invasion of the uterine decidua and decidual vasculature transforms the narrow myometrial spiral arteries into the low-resistance uteroplacental vasculature, which is essential for placenta formation [8]. Thus, maternal factors or impaired trophoblast invasion leading to impaired recasting of the spiral uterine arteries can lead to increased resistance to uterine arterial flow and defective placenta formation, This leads to complications related to PE, fetal growth restriction (FGR), and small for gestational age (SGA) [26–28]. UtA-PI as a common index of vascular impedance, its easy to test, regular testing of this quantitative parameter can identify reduced uterine artery blood flow and abnormal uterine artery remodeling, and identify high-risk pregnant women who may face adverse pregnancy outcomes [8]. Myatt et al. [29] used UtA-PI and resistance index measured by the Doppler technique in a low-risk population before 21 weeks of gestation to predict PE with a sensitivity of 43% and a specificity of 67%, including 78% and 66% for early-onset PE. Wright et al. [30] developed an early pregnancy prediction model based on maternal characteristics, MAP, and UtA-PI, which predicted early onset PE by 90% and 57%, respectively, in a large cohort (57458 normal pregnancies and 1426 PE) at a fixed false positive rate of 10%. Poon et al. [31] combined PlGF and PAPP-A to develop a predictive model for maternal characteristics, MAP, and UtA-PI, with 95.3% and 45.6% predictive values for early-onset and late-onset PE, respectively, at a risk cutoff of 1:200 and a false positive rate of 10.9%. Our results agree with those from similar studies. We more intuitively demonstrated that monitoring UtA-PI values in early pregnancy has a high clinical value.

Nevertheless, our study has shortcomings and some limitations. Our threshold effect analysis yielded large distortions in ORs and confidence intervals, which the author speculated could be due to small total sample sizes while adjusting for multiple covariates, which could lead to model overfitting. However, the author believes that it is still of reported significance, and the discovery of this non-linear relationship provides valuable clues for further exploration of the optimal threshold of UtA-PI in clinical application. This methodological attempt to use a two-segment linear model for threshold effect analysis also provides a valuable analytical idea for similar studies, which can help identify the non-linear relationship between exposure factors and outcomes and is of great significance for an in-depth understanding of complex biomedical relationships. Finally, this study is a correlational analysis, and the conclusions are limited to association rather than causality.

## Conclusion

According to our study, higher UtA-PI values were clearly associated with a higher prevalence of PE, and a non-linear U-shaped relationship existed between UtA-PI and PE incidence. When UtA-PI exceeded the inflection point (1.83), the incidence of PE significantly increased. These results need to be validated in larger sample sizes and different cohorts in the future to ensure the reproducibility and broad applicability of the findings.

## Supporting information

S1 File Data sets for statistical analysis. (XLS)

S2 File 11–13 weeks specimen matched with maternal age and gestational age of collecting specimens. (XLSX)
